# Self-Adaptive Filtering Approach for Improved Indoor Localization of a Mobile Node with Zigbee-Based RSSI and Odometry

**DOI:** 10.3390/s19214748

**Published:** 2019-11-01

**Authors:** Anbalagan Loganathan, Nur Syazreen Ahmad, Patrick Goh

**Affiliations:** School of Electrical & Electronic Engineering, Universiti Sains Malaysia, Nibong Tebal 14300, Penang, Malaysia

**Keywords:** Zigbee, RSSI, wireless-sensor networks, odometry, convex optimization, self-adaptive filtering, indoor localization

## Abstract

This study presents a new technique to improve the indoor localization of a mobile node by utilizing a Zigbee-based received-signal-strength indicator (RSSI) and odometry. As both methods suffer from their own limitations, this work contributes to a novel methodological framework in which coordinates of the mobile node can more accurately be predicted by improving the path-loss propagation model and optimizing the weighting parameter for each localization technique via a convex search. A self-adaptive filtering approach is also proposed which autonomously optimizes the weighting parameter during the target node’s translational and rotational motions, thus resulting in an efficient localization scheme with less computational effort. Several real-time experiments consisting of four different trajectories with different number of straight paths and curves were carried out to validate the proposed methods. Both temporal and spatial analyses demonstrate that when odometry data and RSSI values are available, the proposed methods provide significant improvements on localization performance over existing approaches.

## 1. Introduction

Localization has gained a growing interest among researchers and industries due to technological advancements in wireless sensor networks (WSNs). WSNs are typically made up of a set of small, low-energy devices called sensor nodes, optionally with a base station for monitoring purposes. Localization in a WSN is defined as the task of determining real-time coordinates of the sensor nodes, given that the exact positions of some of the static nodes, also called anchor nodes, are known [1].

The past few decades have witnessed a dramatic increase in the development of indoor localization due to its applicability in various real-time location-based services and smart monitoring systems, particularly in buildings and places where the Global Positioning System (GPS) is denied due to poor satellite reception. Techniques for indoor localization can be generally divided into two broad categories, namely, radio frequency (RF)- and non-RF-based. The latter technique exploits non-RF sensors to estimate the movement of a target node and its distance from other nodes. Examples of these include radar, acoustic, ultrasound, and optical sensors. The RF-based technique, on the other hand, utilizes received signal strength between two sensor nodes to estimate the distance between them, hence it is often termed as the received signal strength indicator (RSSI) method. This method does not require additional hardware for estimation, and is preferable in many cases owing to its penetrability as compared to the non-RF approaches [2].

Several RF technologies have been proposed and tested in the literature for RSSI-based indoor localizations, and the most popular ones are WiFi, Bluetooth Low Energy (BLE), Ultrawide Band (UWB), and Zigbee. Each of them has its own advantages and disadvantages, and its efficacy greatly depends on the nature of applications and hardware performance. WiFi, for instance, has become the simplest solution for localization in buildings with high availability of access points with the highest data-rate transfer and throughput, but at the cost of high power consumption. Hence, it may not be suitable for indoor tracking, particularly when sensor nodes are dependent on batteries to operate. BLE, on the other hand, consumes significantly less power, as the maximum data rate is considerably lower than that of WiFi, but it has been demonstrated to be more accurate than WiFi when used for localization due to better estimation on the RSSI-distance model [3]. Both WiFi and BLE signals, however, are easily perturbed by multipath effects, signal reflections, and even small noise perturbation due to the inherent structure of narrowband radio waves, which makes precise distance measurement more difficult to achieve. To address this problem, UWB radio signals have been designed with very short impulse transmissions to preserve the signal’s integrity and strength, as they are not easily overlapped with noise or multipath effects. The downsides of UWB are, nonetheless, the high cost of the equipment and a requirement for dedicated infrastructure [4]. ZigBee is another Bluetooth-like technology that operates at about a quarter of Bluetooth’s 1 Mbps maximum data rate. The low data rate makes it unsuitable for applications with high-speed data transmission, but the solution enables multiyear battery life and connection to a large number of nodes. A detailed comparison between these technologies can be found in [2,5].

Localizing a mobile node in an indoor environment is relatively more challenging than localizing a static node as it continuously changes its location with time [6]. Moreover, for RSSI-based localizations, a node at some places may only receive two RSSI values at certain time instances while moving due to occlusions from the environment and limitations on the communication modules, such as sudden transceiver failures, or limited energy and bandwidth. This scenario makes it impossible to localize if one is to use traditional triangulation or multilateration-based methods. In order to circumvent this problem, several studies have begun to exploit compressive sensing theory [7] that allows sparse and noisy signals to be recovered with only a small number of RSSI measurements. Via this theory, not only can the number of anchor nodes be minimized [8], it is also possible to localize multiple target nodes at the same time [9]. The work in [9] shows how the compressive-sensing technique can be adopted along with $\ell_{1}$ minimization programs to localize multiple target nodes, where the results are then validated via a number of simulations. In the case where a sufficiently large number of anchor nodes are available, fingerprinting is one of the approaches that has been extensively adopted by many researchers as it is able to construct a radio map from recorded RSSI values during the offline phase [10,11]. Hence, any changes of signal strength during the online localization phase are cross-checked with the stored fingerprints to estimate the best location of the target node, either deterministically [12] or probabilistically [13]. This method is particularly popular for localizing smartphone or mobile-device users in buildings equipped with many WiFi access points. Nonetheless, the major challenges that are always associated with this approach are the inherent noise and large fluctuations of the signal strength due to the time-varying orientation of smartphones or unexpected obstacles between anchors and users. In this regard, several techniques have been proposed such as incorporating artificial-intelligence-based methods and using suitable filters to minimize the influence of multipath effects and interference [14,15,16,17,18,19]. Specific examples include a reliability-augmented particle filter, where the uncertainty of a user’s true step length can be compensated [11], and fusing fingerprints with uncertain mutual distances that are constrained within specific bounds [10].

In the field of mobile robotics, non-RF based devices are typically utilized as proprioceptive sensors in the odometry approach, which is a common technique to localize wheeled mobile robots. While these sensors can estimate the path and pose of the robot relatively more accurate than by using the RSSI-based methods, errors due to wheel slippage, sensor drifts, and other environmental disturbances may inevitably accumulate and eventually deteriorate the tracking performance [20]. To prevent the uncertainties in odometry from growing unbounded, a probabilistic state estimation was introduced which fuses odometry with other exteroceptive sensors such as sonar, lidar, laser range finders, and cameras [21]. The most used algorithms reported in the literature are Markov [22] and Kalman filter localizations [23] where both rely on probabilistic motion and measurement models. Markov localization assumes that the map is known and uses fine-grained and metric discretization of the state space. The configuration space is divided into a number of cells where each cell contains the probability of the robot to be in that cell. During the prediction and update stages, all cells are updated; thus, this method entails high computational costs. The Kalman filter, on the other hand, is computationally less intensive, as the probability distributions of both robot and sensor model are assumed to be Gaussian; hence, only the mean and variance of the distribution need to be updated at each time step [24].

As an alternative to the probabilistic-based method where the optimal solution heavily relies on map accuracy, sensing, and the computational capabilities of the robot, integrating odometry with WSN-based localizations has also become a new trend in recent years [20,25,26,27]. This particular strategy has recently found a wide variety of applications, such as human tracking [14,28,29,30], navigational assistance [5], warehouse management [2,31], and monitoring underground areas in post-disaster environments [32]. In smart buildings where WSN is installed for electrical usage and environmental monitoring purposes [33], this approach can keep operational costs to a minimum as no additional hardware is required to assist the odometry-based localization. The work in [27] shows an example where the RSSI and phase shift of signals from passive UHF-RFID tags, which are installed in the ceiling of the environment, could be combined with odometry data to improve localization performance. Fusing WiFi- or BLE-based RSSI with inertial measurement units (IMUs), which typically consists of an accelerometer and a gyroscope, is also becoming more popular due to the advanced technology of smartphones and mobile devices [23,29,34]. From the WSN perspective, another notable benefit of introducing mobility to the network is that it can increase its capability on various aspects, including flexible topology adjustment and automatic node deployment [35]. In addition, precise and fast localization of mobile nodes in GPS-denied environments allows several crucial tasks to be executed, such as search-and-rescue missions and map-building during emergency cases.

While integrating WSN with odometry localizations can be advantageous for compensating deficiencies from individual methods, suitable fusion techniques must be employed in order to achieve optimal experimental results without sacrificing the desired design requirements. A number of studies suggested adopting the Kalman filter and/or its variants [20,36], while others preferred different approaches due to some disputes over the suitability or effectiveness of the filters particularly for compensating high variability or uncertainties in the signal strength [18,19]. In practice, however, the accuracy, dynamic range of the signals and their variations highly depend on the specific hardware platforms used, the infrastructure impact, as well as spatial or environmental factors, which make any direct comparison between existing methods less conclusive. In other words, while some techniques work tremendously well in a particular environment, this does not mean others are less effective in general. Nonetheless, approaches that have been experimented in the literature often pave the way for future research in and development of WSN technologies.

The focus of this study is on localizing a continuously moving target node using Zigbee-based RSSI measurements and odometry when no knowledge of the map or fingerprint database is available. To the best of the authors’ knowledge, little attention has been paid in the literature to date on investigating and optimizing the indoor-localization technique for a continuously moving node within this scope. Plus, many studies on indoor localizations focused on reducing the steady-state position error. Alternatively stated, when target nodes are in a continuous motion, only their final positions or their positions while they are stationary have been of interest for performance evaluations. In this work, we introduce mobility to the target node by using a nonholonomic wheeled mobile robot with a specific velocity profile. The main contributions of this paper can be stated as follows:

1. a novel approach to compensate for uncertainties in the Zigbee-based RSSI and odometry localizations for a continuously moving target node;
2. a new methodological framework to fuse Zigbee-based RSSI and odometry-based localizations with convex searches on optimal weighting parameters; and
3. a self-adaptive filtering technique that autonomously optimizes the weighting parameter during the target node’s translational and rotational motions which exhibit different but consistent error patterns.

The methods proposed in this work are also computationally less onerous as localization is only corrected using weighting parameters that are updated on the basis of the robot’s rotational velocity. Several real-time experiments consisting of four different trajectories with different numbers of straight paths and curves were carried out to validate the proposed techniques. Both temporal and spatial analyses demonstrated that, when odometry data and RSSI values are available, the proposed methods provide significant improvements on localization performance over existing approaches that also include the extended Kalman filter. In addition, we also showed that the new self-adaptive filtering technique which optimizes the error minimizer when the rotational velocity within a specified range is detected considerably outperformed the rest.

The remainder of the paper is organized as follows: Section 2 explains the indoor localization framework and strategies, which begins with a brief description on the system architecture and the path-loss propagation model, followed by the RSSI-based localization method, odometry, and the main proposed strategies involving convex optimizations when both the RSSI-based method and odometry are hybridized. Section 3 presents the experimental results, qualitative and quantitative performance evaluations and discussions. Results of the proposed methods are then concluded and further discussed in Section 4 with future works. For notations and abbreviations that are frequently used in this manuscript, readers are referred to the last section before the references.

## 2. Indoor Localization Framework and Strategies

### 2.1. System Architecture and Path-Loss Propagation Model

Zigbee is a low-cost wireless communication technology based on the IEEE 802.15.4 standard, which is often used to create personal area networks. Due to its low power consumption and secure networking capabilities, it is often preferable for wireless control and monitoring in smart-building technologies.

**Figure 1:**
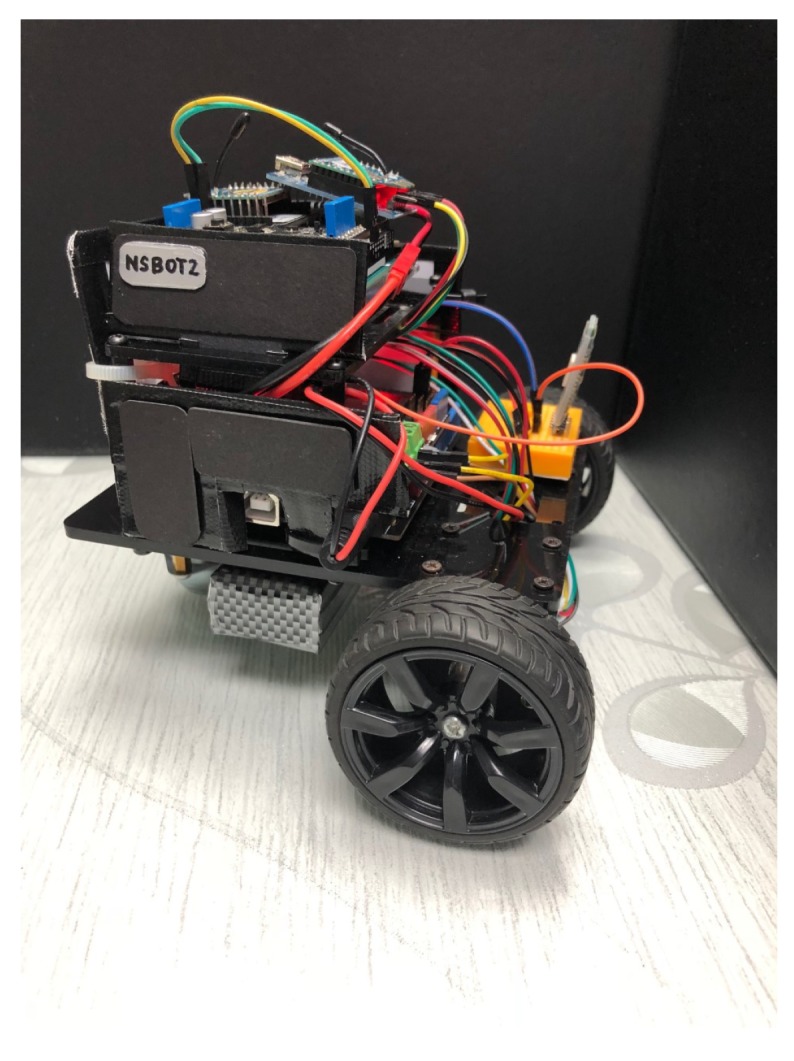
NSBot2: A nonholonomic wheeled mobile robot equipped with ATMega microcontrollers and Zigbee radio modules with IEEE 802.15.4 standard.

In this study, we use XBee embedded modules with the Zigbee protocol to create four transmitters that are placed at preassigned positions, and one receiver which is the target node to be localized. In order to introduce mobility to the target node and to verify the proposed indoor localization strategies, NSBot2, which is a nonholonomic wheeled mobile robot was built with ATMega microcontrollers connected to the target node as shown in Figure 1.

With regard to control mechanism, NSbot2 was preprogrammed with closed-loop cascade control where its motion was constrained to constant translational and rotational velocities. This technique does not just allow the robot to travel along various types of planned path, but it also enables each experiment with a specific path to be repeated with minimal variations.

The basic idea for indoor localization with RSSI is that the target node to be localized stays at a fixed coordinate, and the RSSI from different anchor nodes with known positions are measured. RSSI values can be converted into distances using a path-loss model [37], which are then used with the triangulation, multilateration, or trilateration method to estimate the position of the target node [38].

One of the most commonly used path-loss propagation models in a WSN is the log-distance function. Via this model, the value of RSSI, β (in dBm) can be theoretically expressed as follows [39]:

(1) $$\begin{array}{c} \beta \left(d\right)=\beta_{0}-10\gamma \log_{10}\left(\frac{d}{d_{0}}\right) \end{array}$$

where *d* (in m) is the distance between transmitter (anchor node) and receiver (target node), $d_{0}$ is the free space reference distance, typically 1 m, γ is the path-loss exponent coefficient, and $\beta_{0}$ is the RSSI value when the transmitter and the receiver are 1 m apart, which in this case is found to be −38 dBm. The theoretical value for γ with some calibrations is 1.8. From Equation (1), the distance *d* can be simply retrieved with:

(2) $$d\left(\beta \right)=d_{0}\times 10^{\frac{\beta_{0}-\beta }{10\gamma }}.$$

**Figure 2:**
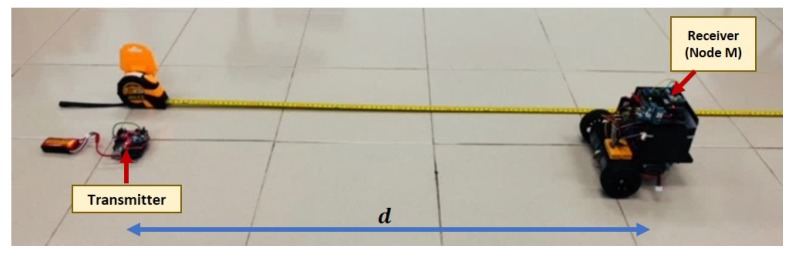
Experimental setup to estimate real path-loss model of the mobile node.

In real situations, when the target node is continuously moving, the effects of noise from multipath propagation, signal occlusions, and diffractions can be undesirably amplified. Hence, the theoretical RSSI-distance relation in Equation (1) may no longer be sufficient to predict the actual path-loss model. In order to estimate the real distance for a continuously moving node more accurately, sampled data for β are collected when the transmitter is placed at a fixed coordinate, and the receiver, named Node M, is moved at a speed of approximately 20 cm/s from d=100 cm until d=500 cm. This is illustrated in Figure 2.

A curve-fitting method is then utilized to generate an improved path-loss propagation model, $\beta_{f}$, as follows:

(3) $$\begin{array}{cc} g= & \frac{n\sum_{i=1}^{n}\left(\beta_{i}\log_{10}q_{i}\right)-\sum_{i=1}^{n}\beta_{i}\sum_{i=1}^{n}log_{10}q_{i}}{n\sum_{i=1}^{n}{\left(\log_{10}q_{i}\right)}^{2}-{\left(\sum_{i=1}^{n}\log_{10}q_{i}\right)}^{2}},\\ \; & h=\frac{\sum_{i=1}^{n}\beta_{i}-g\sum_{i=1}^{n}\left(\log_{10}q_{i}\right)}{n},\\ \; & \;\beta_{f}\left(q\right)=h+g\log_{10}\left(q\right). \end{array}$$

where $q=d/d_{0}$; and *n* is the number of sampled data.

### 2.2. RSSI-Based Localization

In this work, four transmitters were configured as anchor nodes (Anchors A, B, C, and D); hence, four distance values can be obtained at each time instance from the path-loss propagation model. They are also placed at points where any three of them are always noncollinear, which ensures the feasibility of the trilateration method at each time instance.

Consider (x,y) as the unknown coordinate of the mobile node, and $\left(x_{j},y_{j}\right)$ with j=1,2,3 be the first three coordinates from three different anchor nodes received by the mobile node. Let $d_{j}$ be the estimated distance from Node M to $\left(x_{j},y_{j}\right)$, and

(4) $$\begin{array}{c} \left[\begin{array}{cc} \alpha_{1} & \gamma_{1}\\ \alpha_{2} & \gamma_{2} \end{array}\right]\left[\begin{array}{c} x\\ y \end{array}\right]=\left[\begin{array}{c} \delta_{1}\\ \delta_{2} \end{array}\right] \end{array}$$

where

(5) $$\begin{array}{cc} \alpha_{1} & =-2x_{1}+2x_{2},\;\alpha_{2}=-2x_{2}+2x_{3},\\ \gamma_{1} & =-2y_{1}+2y_{2},\;\gamma_{2}=-2y_{2}+2y_{3},\\ \delta_{1} & =d_{1}^{2}-d_{2}^{2}+x_{2}^{2}-x_{1}^{2}+y_{2}^{2}-y_{1}^{2},\\ \delta_{2} & =d_{2}^{2}-d_{3}^{2}+x_{3}^{2}-x_{2}^{2}+y_{3}^{2}-y_{2}^{2}. \end{array}$$

The coordinate of the mobile node can then be retrieved via Cramer’s rule as follows:

(6) $$\begin{array}{c} x=\frac{\left|\begin{array}{cc} \delta_{1} & \gamma_{1}\\ \delta_{2} & \gamma_{2} \end{array}\right|}{\left|\begin{array}{cc} \alpha_{1} & \gamma_{1}\\ \alpha_{2} & \gamma_{2} \end{array}\right|}=\frac{\delta_{1}\gamma_{2}-\delta_{2}\gamma_{1}}{\gamma_{2}\alpha_{1}-\gamma_{1}\alpha_{2}},\;y=\frac{\left|\begin{array}{cc} \alpha_{1} & \delta_{1}\\ \alpha_{2} & \delta_{2} \end{array}\right|}{\left|\begin{array}{cc} \alpha_{1} & \gamma_{1}\\ \alpha_{2} & \gamma_{2} \end{array}\right|}=\frac{\alpha_{1}\delta_{2}-\alpha_{2}\delta_{1}}{\gamma_{2}\alpha_{1}-\gamma_{1}\alpha_{2}}. \end{array}$$

### 2.3. Odometry

**Figure 3:**
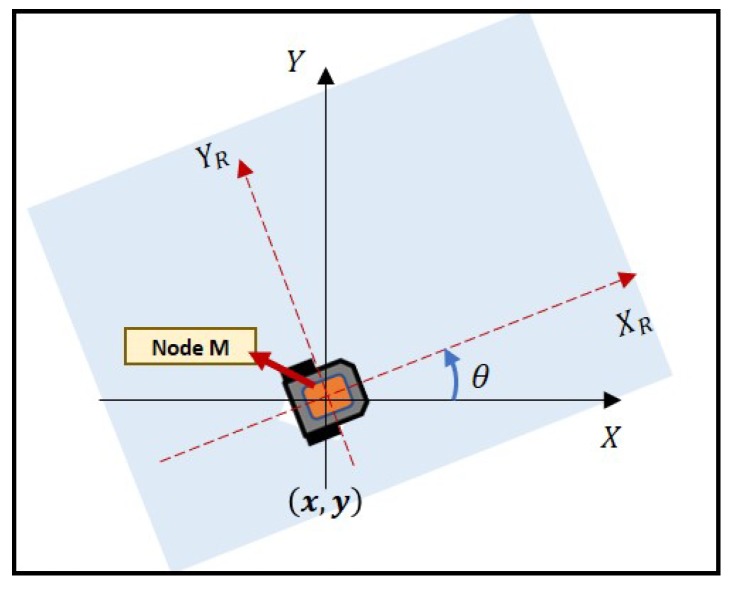
Overview of mobile robot in [X,Y] and $\left[X_{R},Y_{R}\right]$ frames.

Odometry is a process of estimating the position change of a mobile robot on the basis of data from motion sensors. For this research, the encoders on both wheels of the mobile robot were used to estimate the path taken as well as the heading direction. Figure 3 illustrates the robot’s position with respect to two different frames, namely [X,Y] and $\left[X_{R},Y_{R}\right]$. [X,Y] denotes the world coordinate system, whereas $\left[X_{R},Y_{R}\right]$ represents the robot-attached frame. The robot’s translational velocity, *v*, and rotational velocity, ω, can be written as follows [24]:

(7) $$\begin{array}{cc} v & =\frac{(\omega_{r}+\omega_{l})r}{2}, \end{array}$$

(8) $$\begin{array}{cc} \omega & =\frac{(\omega_{r}-\omega_{l})r}{D}, \end{array}$$

where *r* is the radius of the wheel, *D* is the azimuth length between wheels, and $\omega_{r}$ and $\omega_{l}$ represent the angular velocities of the right and left wheels respectively. Let $\left(x_{c},y_{c}\right)$ be the center of the robot, $\left(x_{n},y_{n}\right)$ be the current coordinate based on the $\left[X_{R},Y_{R}\right]$ plane, (x,y) be the current coordinate based on the [X,Y] plane, and θ be the robot’s heading angle with respect to the *X* axis. The relation between (x,y), $\left(x_{n},y_{n}\right)$, and $\left(x_{c},y_{c}\right)$ can be written as:

(9) $$\begin{array}{c} \left[\begin{array}{c} x\\ y \end{array}\right]=J\left(\theta \right)\left[\begin{array}{c} x_{n}\\ y_{n} \end{array}\right]+\left[\begin{array}{c} x_{c}\\ y_{c} \end{array}\right] \end{array}$$

where

(10) $$\begin{array}{c} J\left(\theta \right)=\left[\begin{array}{cc} \cos\theta & -\sin\theta \\ \sin\theta & \cos\theta \end{array}\right]. \end{array}$$

The movement of the robot can be decomposed into linear velocities with respect to the *X* and *Y* axes, i.e., $\dot{x}$ and $\dot{y}$, which have a relation with linear and angular velocities as follows:

(11) $$\begin{array}{c} \left[\begin{array}{c} \dot{x}\\ \dot{y}\\ \dot{\theta } \end{array}\right]=\left[\begin{array}{cc} \cos\theta & 0\\ \sin\theta & 0\\ 0 & 1 \end{array}\right]\left[\begin{array}{c} v\\ \omega \end{array}\right]. \end{array}$$

Let $f\left(x,y,\theta \right)=\left(x_{0},y_{0},\theta_{0}\right)$ be the previous position of the robot. When the robot is only driving forward in the $X_{R}$-axis direction, only translational velocity *v* is non-zero, and $\theta =\theta_{0}$. Hence, we have $\dot{x}=v\cos\theta_{0}$ and $\dot{y}=v\sin\theta_{0}$. The current update on *f* is then obtained by integrating $\dot{x}$ and $\dot{y}$, which leads to

$$\begin{array}{cc} x & =vt_{s}\cos\theta_{0}+x_{0},\\ y & =vt_{s}\sin\theta_{0}+y_{0}, \end{array}$$

where $t_{s}$ is the sampling time. When the robot steers, the trajectory is a circular arc with a radius ρ=v/ω, i.e.,

(12) $$\rho =\frac{D(\omega_{r}+\omega_{l})}{2(\omega_{r}-\omega_{l})},$$

The current heading angle of the robot can be calculated as $\theta =\int_{0}^{t_{s}}\omega dt=\omega t_{s}+\theta_{0}$. The position of the robot can therefore be estimated with

(13) $$\begin{array}{c} \left[\begin{array}{c} x\\ y\\ \theta \end{array}\right]=\left[\begin{array}{cc} J(\theta ) & 0\\ 0 & 1 \end{array}\right]\left[\begin{array}{c} \rho \sin(\omega t_{s})\\ \rho (1-\cos\left(\omega t_{s}\right))\\ \omega t_{s} \end{array}\right]+\left[\begin{array}{c} x_{0}\\ y_{0}\\ \theta_{0} \end{array}\right]. \end{array}$$

### 2.4. Improved Localization Techniques

**Figure 4:**
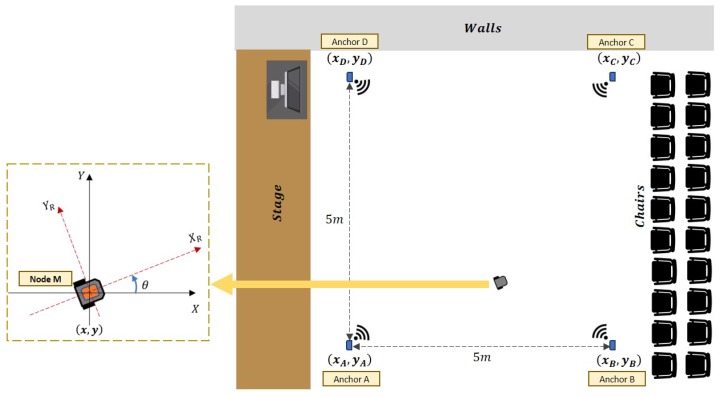
Illustration of the experimental setup from top view (not to scale).

Both the RSSI- and odometry-based localization methods have their own advantages and disadvantages. With regard to the RSSI method, a notable stumbling block when operating indoors is signal-strength attenuation that occurs via various mechanisms such as diffraction, reflection, and scattering due to environmental obstacles like walls. Odometry, on the other hand, is susceptible to measurement errors that are typically caused by wheel slip and sensor drift. To compensate for the deficiencies of individual methods, a preliminary set of experiments were conducted to investigate the accuracy of each method and to search for optimal weighting parameters before both localization techniques are fused. Figure 4 illustrates the experimental setup where the four anchor nodes are placed at the vertices of a square with

$$\begin{array}{c} \left(x_{A},y_{A}\right)=\left(-1,-1\right),\;\left(x_{B},y_{B}\right)=\left(4,-1\right),\;\left(x_{C},y_{C}\right)=\left(4,4\right),\;\mathrm{and}\;\left(x_{D},y_{D}\right)=\left(-1,4\right). \end{array}$$

As our focus was on localizing a continuously moving robot, we took into account the transient error defined by

(14) $$\begin{array}{c} e\left(t\right)=\sqrt{{\left(x\left(t\right)-x_{r}\left(t\right)\right)}^{2}+{\left(y\left(t\right)-y_{r}\left(t\right)\right)}^{2}}, \end{array}$$

where (x,y) represents the observed coordinate of the robot, while $x_{r}\left(t\right)$ and $y_{r}\left(t\right)$ indicate the actual position of the robot in the x and y directions respectively, which were obtained by using a camera. The localization error for the whole trajectory was then defined as

(15) $$\begin{array}{c} E_{T}\left(x,y\right)=\int_{t_{0}}^{t_{f}}e\left(\tau \right)d\tau , \end{array}$$

where $t_{0}$ and $t_{f}$ are the initial and final execution time respectively. To increase the localization accuracy of the RSSI-based method, we propose the following lemma, which is based on the improved path-loss model and a recursive implementation of moving average filter:

**Lemma** **1.** *Let* $\beta_{a},\beta_{b},\beta_{c}$ *, and* $\beta_{d}$ *be the RSSI values based on the path-loss model estimated by Equation *(3)* for Anchors A, B, C, and D, respectively, and R be the number of points in the average that is selected a priori. Given*

(16) $$\beta_{r}\left(0\right),\beta_{r}\left(1\right),\ldots ,\beta_{r}\left(R-1\right),\;r=a,b,c,d$$

*and*

(17) $${\tilde{\beta }}_{r}\left(R\right)=\frac{1}{R}\sum_{k=0}^{R-1}\beta_{r}\left(k\right),\;r=a,b,c,d,$$

*the following equations are then equivalent:*

(18) $${\tilde{\beta }}_{r}\left(i\right)=\frac{1}{R}\sum_{j=1}^{R}\beta_{r}\left(i-j\right),\;,i\geq R+1,\;r=a,b,c,d$$

(19) $${\tilde{\beta }}_{r}\left(i\right)={\tilde{\beta }}_{r}\left(i-1\right)+Q\beta_{r}\left(i-1\right)-Q\beta_{r}\left(i-\left(R+1\right)\right)\;,i\geq R+1,\;Q=1/R,\;r=a,b,c,d$$

**Proof.**  From Equation (18), we have

(20) $${\tilde{\beta }}_{r}\left(i\right)=Q\left[\beta_{r}\left(i-1\right)+\beta_{r}\left(i-2\right)+\ldots +\beta_{r}\left(i-\left(R-1\right)\right)+\beta_{r}\left(i-R\right)\right].$$

It also follows that

(21) $${\tilde{\beta }}_{r}\left(i-1\right)=Q[\beta_{r}\left(i-2\right)+\beta_{r}\left(i-3\right)+\ldots +\beta_{r}\left(i-R\right)+\beta_{r}\left(i-\left(R+1\right)\right].$$

Equation (20) can then be restructured into

(22) $${\tilde{\beta }}_{r}\left(i\right)={\tilde{\beta }}_{r}\left(i-1\right)-Q\beta_{r}\left(i-\left(R+1\right)\right)+Q\beta_{r}\left(i-1\right),$$

which is also equivalent to Equation (19). □

If the values of $\beta_{r}$ are subject to Gaussian noise, the effects from the noise can be suppressed via Equation (19) in Lemma 1, which consequently leads to a smoother localization when $d({\tilde{\beta }}_{r})$, which represents the Euclidean distance from Node M to the respective anchor node (calculated using Equation (2)) that is used with the trilateration method in Equations (4)–(6). The recursive filtering method in Equation (19) allows fast computation for real-time implementation which is desirable in our case as the target node considered is continuously moving.

Throughout the paper, we write $\beta_{T}$ as the localization via the theoretical path-loss model as in Equation (1) with the filtering technique in Equation (19), $\beta_{N}$ as the localization based on Lemma 1, and $O_{d}$ as the localization based on the odometry approach as described in Section 2.3. The localization technique using raw RSSI values with the theoretical path-loss model alone was omitted in this work due to its worse performance.

**Figure 5:**
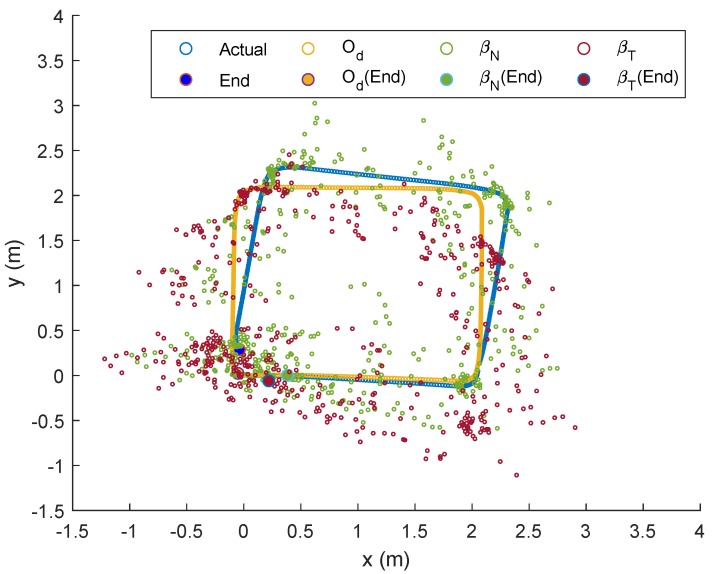
Spatial-localization error analysis in XY-plane.

For the preliminary experiment, the robot was initially placed at (0,0), and moved in a square-like trajectory with three turns until it reached its initial position again. The improved path-loss model for each anchor node via Equation (3) gave $\left(h_{A},h_{B},h_{C},h_{D}\right)=\left(-38.2,-38.9,-38.4,-37.0\right)$ and $\left(g_{A},g_{B},g_{C},g_{D}\right)=\left(-8.39,-8.91,-9.65,-11.6\right)$, where $h_{i}$ and $g_{i}$ indicate the values of *h* and *g* for Anchor *i*, respectively. The robot’s trajectories based on $O_{d}$, $\beta_{T}$ and $\beta_{N}$ (with *R* set to 5) as well as the actual trajectory from the preliminary experiment, are outlined in Figure 5.

An immediate observation from the figure is that the actual trajectory is slightly skewed compared to the path estimated from $O_{d}$ after the first turn (approximately at $\left(x_{r},y_{r}\right)=\left(2,0\right)$), and the localization error becomes higher after the second turn, which then accumulates until the robot stops. Trajectories based on $\beta_{N}$ and $\beta_{T}$, on the other hand, were clearly worse than the odometry approach, as can be seen from the scattered plots. Nevertheless, both showed an interesting pattern, whereby plots were less scattered during instances when the robot steered at each turn, and relatively more scattered when the robot moved in a straight line. A possible justification for this is that less noise is captured when Node M stays within a place for more than a threshold of time. In other words, localization errors based on RSSI methods are minimal when the mobile node does not move too fast.

**Figure 6:**
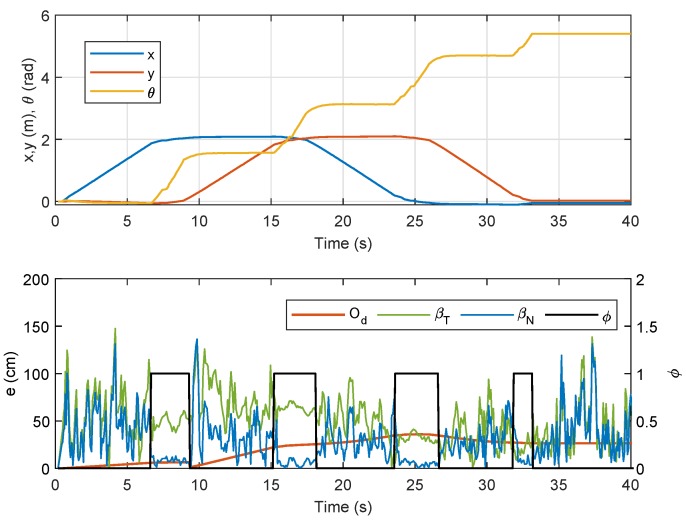
Position of NSBot2 in terms of x,y and θ based on odometry (**top**); temporal localization-error analysis (**bottom**).

In order to investigate the response of e(t) when the robot steers, we introduce an indicator variable, ϕ, which takes the value of 1 when the rotational velocity goes beyond a specified threshold, and 0 otherwise. This can be mathematically described by

(23) $$\begin{array}{cc} \phi & =\left\{\begin{array}{cc} 0 & \;\mathrm{if}\;|\omega |\leq \epsilon_{w}\\ 1 & \;\mathrm{if}\;|\omega |>\epsilon_{w} \end{array},\right. \end{array}$$

where the threshold value $\epsilon_{w}=0.1$ rad/s was selected to avoid capturing any small temporary steering motion during translational movement. Temporal data for the localization error analysis with respect to ϕ are shown in Figure 6. From the figure, it is clearly seen that localization error from $\beta_{T}$ was, in most instances, higher than that from $\beta_{N}$ and $O_{d}$. Another observation is that, although the error from $O_{d}$ was generally lower than that from $\beta_{T}$ and $\beta_{N}$, the error from $\beta_{N}$ was either almost similar or lower than that of $O_{d}$ when ϕ=1. Numerical analysis on $E_{T}$ for all the three methods is recorded in Table 1 for a set of three tests, and it is shown that $E_{T}$ from $\beta_{N}$ was the lowest when ϕ=1, even though $O_{d}$ beats the rest in terms of total $E_{T}$.

Preliminary analysis of the above localization error indicates that performance can be improved by hybridizing $O_{d}$ and $\beta_{N}$, and may be further enhanced by considering the value of ϕ. This, however, requires a search on the optimal weighting parameters that can minimize the localization error when both $O_{d}$ and $\beta_{N}$ are hybridized. To this end, the following proposition is introduced:

**Proposition** **1.** *Define*

(24) $$\begin{array}{c} \left[\begin{array}{c} x(t)\\ y(t) \end{array}\right]=\left[\begin{array}{cc} \mu & 1-\mu \\ \mu & 1-\mu \end{array}\right]\left[\begin{array}{cc} x_{1}\left(t\right) & y_{1}\left(t\right)\\ x_{2}\left(t\right) & y_{2}\left(t\right) \end{array}\right], \end{array}$$

*where* $\left(x_{1},y_{1}\right)$ *and* $\left(x_{2},y_{2}\right)$ *correspond to the estimated coordinates obtained from* $\beta_{N}$ *and* $O_{d}$ *respectively. If there exists*

(25) $$\begin{array}{c} \mu_{0}^{*}=\arg\min_{\mu \in [0,1]}\int_{t_{0}}^{t_{f}}{\left[{\left(x\left(\tau \right)-x_{r}\left(\tau \right)\right)}^{2}+{\left(y\left(\tau \right)-y_{r}\left(\tau \right)\right)}^{2}\right]}^{1/2}d\tau \end{array}$$

*where* $\left(x_{r},y_{r}\right)$ *denotes the reference coordinate, then the new estimated coordinate* (x,y) *of the mobile node with* $\mu =\mu_{0}^{*}$ *has a smaller localization error than that from* $O_{d}$ *and/or* $\beta_{N}$ *alone.*

**Proof.**  The integral term on the right-hand side of Equation (25) corresponds to localization error, $E_{T}$ from Equations (14) and (15) when μ=0, $E_{T}$ reduces to that of $O_{d}$, whereas when μ=1, $E_{T}$ reduces to that of $\beta_{N}$. Hence, the existence of $\mu_{0}^{*}$ is also equivalent to the existence of a minimum $E_{T}$ in the search space of μ. □

**Figure 7:**
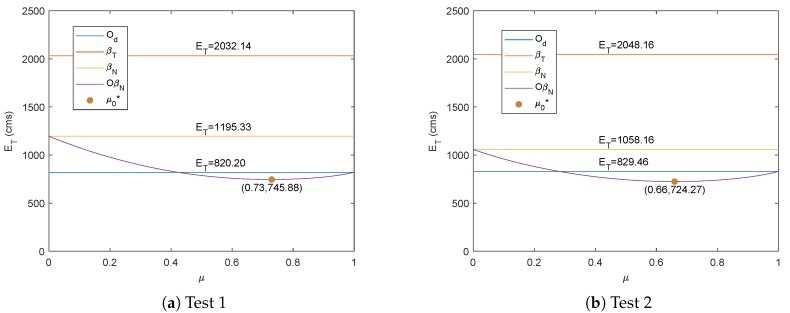

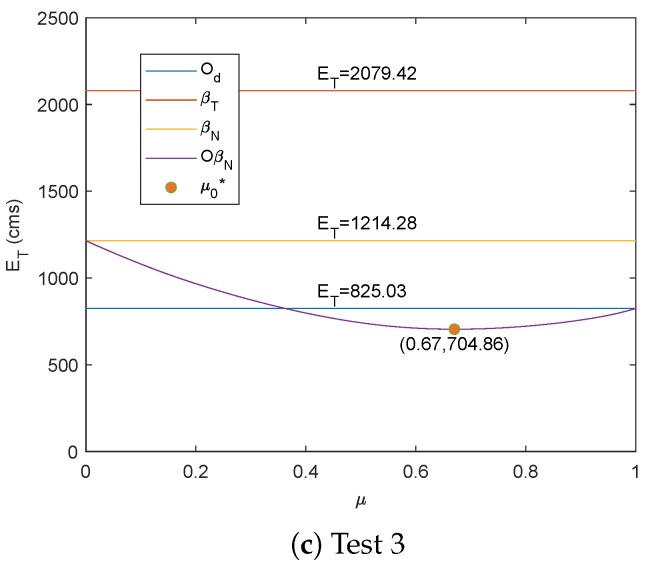
Optimal parameter of μ from Proposition 1 for three tests.

Figure 7 shows the resulting $E_{T}$ which is represented by $O\beta_{N}$ when μ from Equation (24) is varied between its minimum and maximum values for the three tests. Due to the convexity of the search, the minimum value of $E_{T}$ can be attained for each case with weighting parameter $\mu =\mu_{0}^{*}$ which has a variance of less than 0.1. It is also worth to note that μ and 1−μ correspond to the weighting parameters for $\beta_{N}$ and $O_{d}$, respectively, hence $\mu_{0}^{*}$ acts as the error minimizer when both methods are combined.

In order to boost te localization performance, the following corollary is proposed by making the result in Proposition 1 self-adaptive to the value of ϕ.

**Corollary** **1.** *Let*(x,y)*be defined as in Equation *(24)*,*$t_{f}$*be the final execution time, and*

(26) $$\begin{array}{c} \mu_{1}^{*}=\arg\min_{\mu \in [0,1]}\sum_{k=1}^{k_{f}}\int_{t_{k0}}^{t_{kf}}{\left[{\left(x\left(\tau \right)-x_{r}\left(\tau \right)\right)}^{2}+{\left(y\left(\tau \right)-y_{r}\left(\tau \right)\right)}^{2}\right]}^{1/2}d\tau \end{array}$$

*where k is the k-th time*ϕ=1*during*$t\in [0,t_{f}]$, $k_{f}$*is the total number of times*ϕ=1*, and*$t_{kf}-t_{k0}$*is the duration when*ϕ=1*for the k-th time*ϕ=1*. If Equation *(26)* is feasible, the localization performance can then be further improved by setting*$\mu =\mu_{0}^{*}$whenϕ=0*, and*$\mu =\mu_{1}^{*}$whenϕ=1*.*

**Proof.**  If Equation (26) is feasible, then the total localization error from the estimated new coordinate (x,y) during ϕ=1 is smaller than that from $O_{d}$ and $\beta_{N}$ when $\mu =\mu_{1}^{*}$. Hence, combining with the error when $\mu =\mu_{0}^{*}$ whenever ϕ=0 results in a smaller total error than that when $\mu =\mu_{0}^{*}$ for any value of ϕ. □

**Figure 8:**
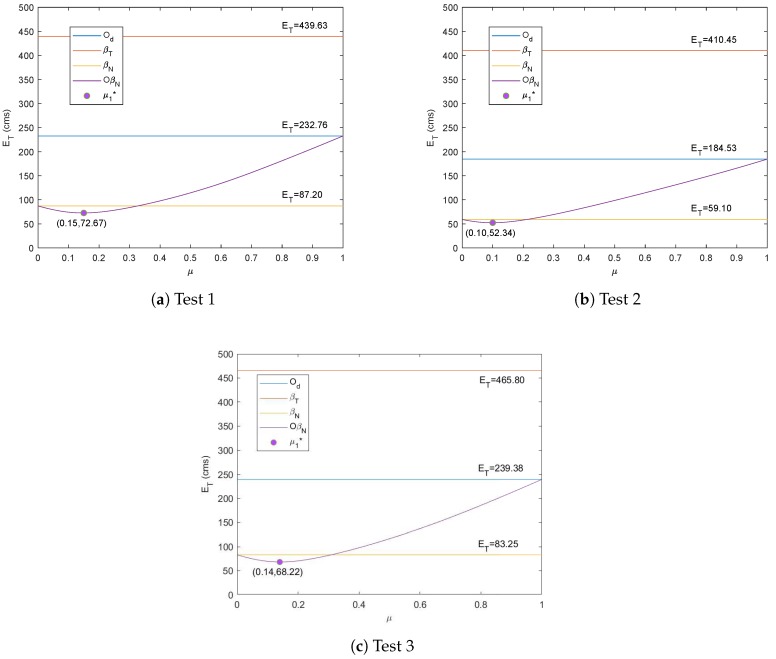
Optimal parameter of μ from Corollary 1 when ϕ=1 for three tests.

The resulting values of $E_{T}$ when ϕ=1 (represented by $O\beta_{N}$) for the three tests are shown in Figure 8. From the figure, it is observed that the minimum values of $E_{T}$ could be obtained with the minimizer $\mu_{1}^{*}$ where its variance is also less than 0.1. The values of $\mu_{0}^{*}$ and $\mu_{1}^{*}$ for all the three tests, along with the corresponding $E_{T}$ from Proposition 1 and Corollary 1, are recorded in Table 2. Compared to the minimum values of $E_{T}$ in Table 1, which were obtained via $O_{d}$ method alone, the resulting $E_{T}$ from Proposition 1 and Corollary 1 were significantly reduced for each test. It is also clear that the best performance was achieved via Corollary 1 due to the introduced self-adaptive filtering method, which varies the error minimizer according to the value of ϕ.

The next section presents a new set of experiments with various trajectories where Proposition 1 and Corollary 1 are applied with the mean values of $\mu_{0}^{*}$ and $\mu_{1}^{*}$ from Table 2.

## 3. Experiment Results, Performance Evaluation, and Discussion

**Figure 9:**
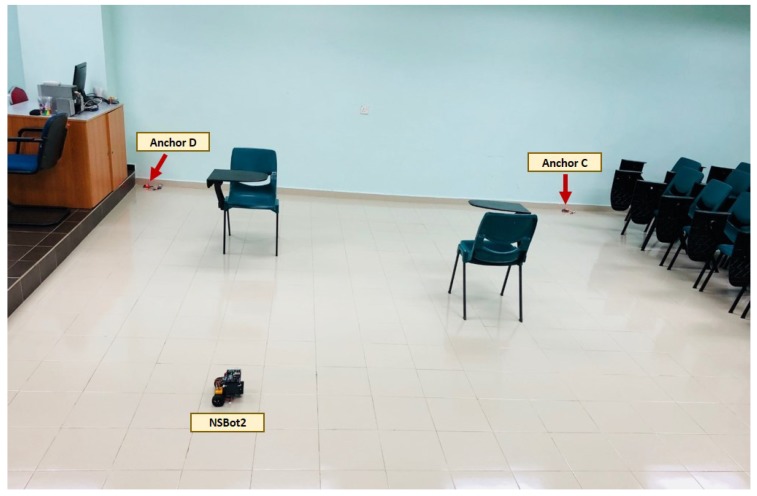
Overview of the experimental setup for performance evaluations. Chairs were randomly placed in the experimental area.

For the new set of experiments, localization performance was evaluated on four different trajectories (Trajectories 1–4) with different numbers of straight paths and curves. For each trajectory, two objects (chairs) were randomly placed in the experimental area as illustrated in Figure 9, and the value of *R* was increased to 8 for both $\beta_{T}$ and $\beta_{N}$ to compensate for the increased multipath effects. Results obtained by Lemma 1, Proposition 1, and Corollary 1 were then compared with those from the theoretical path-loss model, as well as the extended Kalman filter approach, which is an existing method from the literature for sensor fusion. For clarity purposes, we used $O\beta_{p}$ to denote Proposition 1, $O\beta_{c}$ to denote Corollary 1, and $EK_{f}$ to represent the extended Kalman filter.

**Figure 10:**
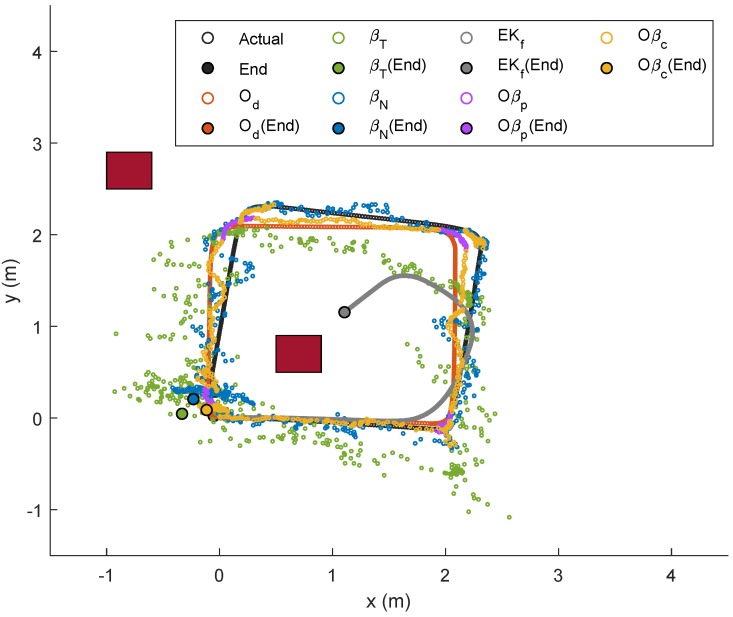
Localization of Node M in the XY-plane for Trajectory 1 experiment.

**Figure 11:**
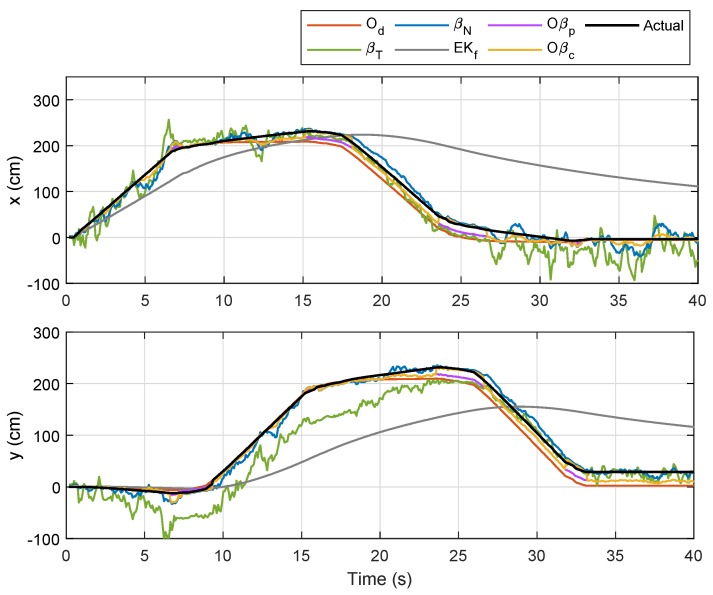
Localization of Node M in *x*- and *y*- directions for Trajectory 1.

**Figure 12:**
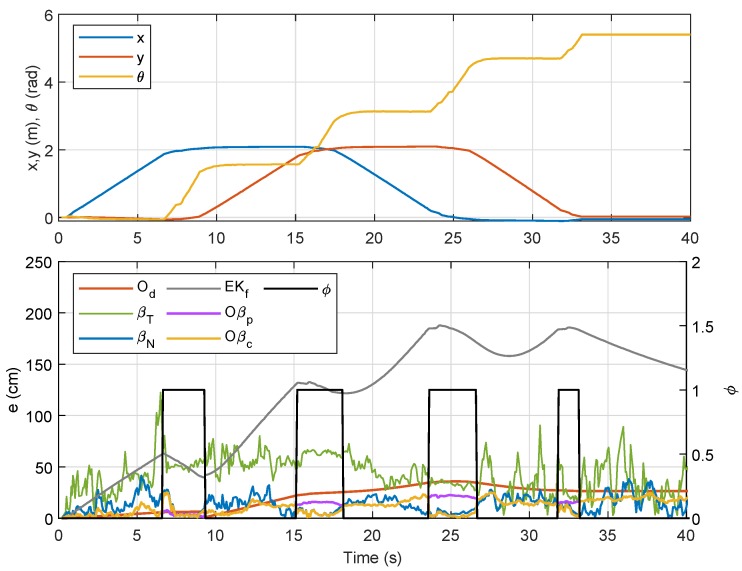
Trajectory 1: Position of NSBot2 in terms of x,y and θ based on odometry (**top**); temporal localization-error analysis (**bottom**).

For the first trajectory, i.e., Trajectory 1, a similar path as in the preliminary experiment was selected, and the localizations of Node M in the XY-plane via the aforementioned techniques were plotted with different colors as indicated by the legends in Figure 10. The corresponding temporal analysis on localizations of Node M in *x*- and *y*-directions is presented in Figure 11, while the corresponding error along with the x,y and θ positions of the robot (based on odometry) are shown in Figure 12. The mobile robot was initially placed at (0,0), and chairs were placed at locations as indicated by the maroon boxes. As seen in Figure 10, localizations that were only based on the RSSI methods (i.e., $\beta_{T}$ and $\beta_{N}$) were relatively more scattered as compared to the other four techniques. The $EK_{f}$ method, despite showing a smooth trajectory (grey line), resulted in localization that was relatively too far from the actual position, which can also be clearly seen in Figure 11. It was also observed that $O\beta_{p}$ and $O\beta_{c}$ gave better location estimation for Node M from its initial until final positions. A much closer estimation to the actual position can be seen via $O\beta_{c}$, particularly when the robot steered at each edge of the trajectory.

Referring to the bottom plot of Figure 12, errors due to $O_{d}$ and $O\beta_{p}$ in the first rotational motion (ϕ=1) were much smaller as compared to errors due to other methods. However, in the second, third, and fourth rotational movements, the error from $O_{d}$ was considerably larger than those from $\beta_{N}$, $O\beta_{p}$, and $O\beta_{c}$, which was a consequence from accumulated errors due to wheel slippage after each turn. In order to provide better evaluation, the experiment for Trajectory 1 was repeated five times, and the numerical results on localization error for each method are compared in Table 3. From the table, the sensor-fusion method via $EK_{f}$ recorded the highest error, while $O\beta_{c}$ recorded the lowest. Besides that, a positive impact can be seen from the result of $\beta_{N}$ where the error was marginally lower than that from $O_{d}$ due to the increased value of the filter coefficient *R* from Lemma 1 for this experiment. We can also conclude that $\beta_{N}$ provides a huge improvement over $\beta_{T}$ for the comparison between RSSI-based methods, and fusing $\beta_{N}$ with $O_{d}$ as described in Proposition 1 (i.e., $O\beta_{p}$) significantly reduces the localization error. A further error reduction was accomplished through $O\beta_{c}$, which prioritized $\beta_{N}$’s localization at each turn via the self-adaptive filtering approach.

**Figure 13:**
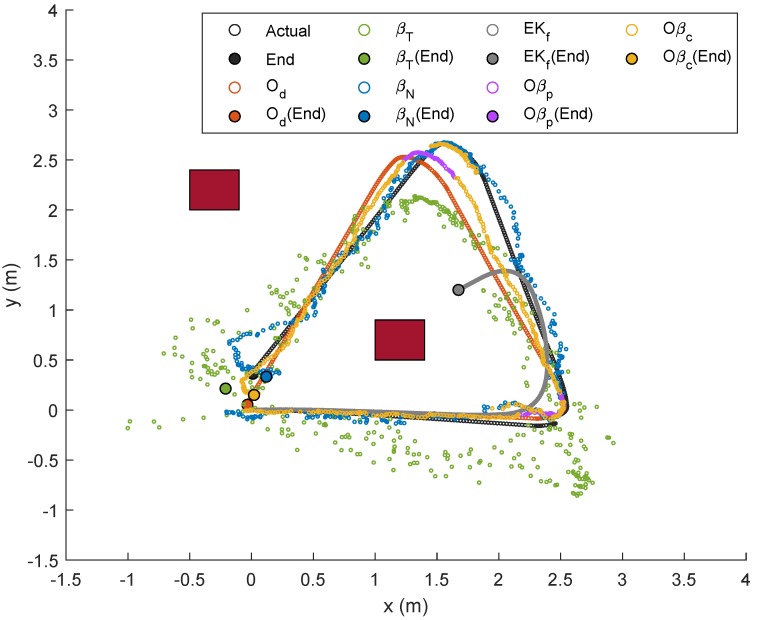
Localization of Node M in xy-plane in Trajectory 2.

**Figure 14:**
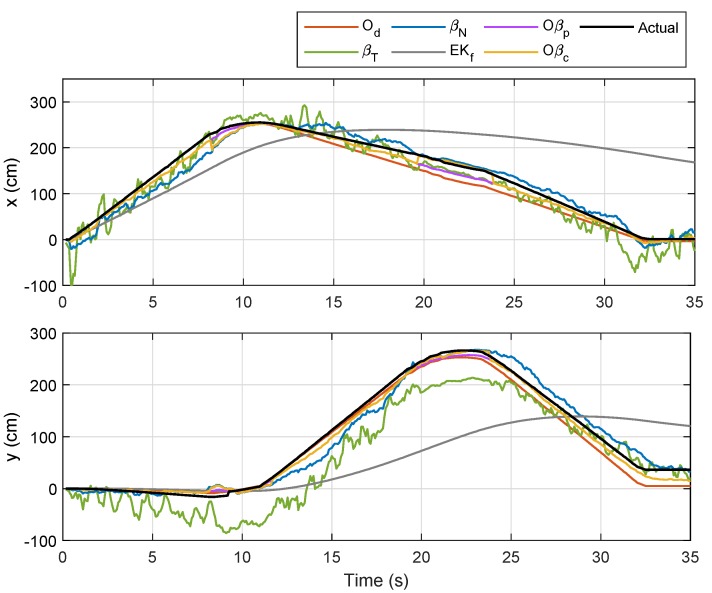
Localization of Node M in *x*- and *y*-directions in Trajectory 2.

**Figure 15:**
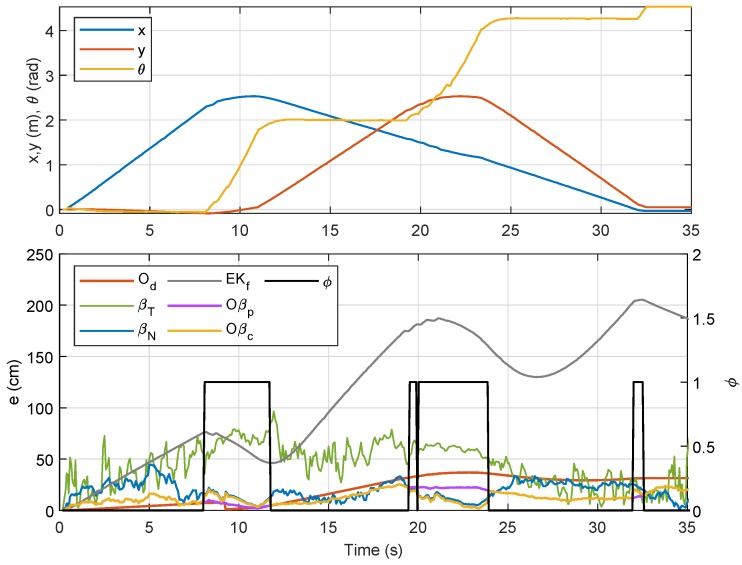
Trajectory 2: Position of NSBot2 in terms of x,y and θ based on odometry (**top**); and temporal localization-error analysis (**bottom**).

For Trajectory 2, which follows a triangle-like path, the corresponding localizations and error plots are depicted in Figure 13, Figure 14 and Figure 15, while the numerical results are presented in Table 4. Although there were only three curves involved, the same trend could be observed whereby $O_{d}$ and $O\beta_{p}$ showed smaller errors than the rest in the first rotational motion, but the error due $O_{d}$ ratcheted up afterwards as it was accumulated. In this case, however, the duration during the rotational motion was slightly smaller than that from the first trajectory; hence, competitive performance between $O_{d}$ and $\beta_{N}$ could be seen from the numerical results of Tests 1–5. Comparing the average errors in Table 4, the method with the worst performance appeared to be similar to that from the first trajectory, which was $EK_{f}$, followed by $\beta_{T}$, $\beta_{N}$, and $O_{d}$. Both $O\beta_{p}$ and $O\beta_{c}$, on the other hand, exhibited the same performance as in the previous experiment.

**Figure 16:**
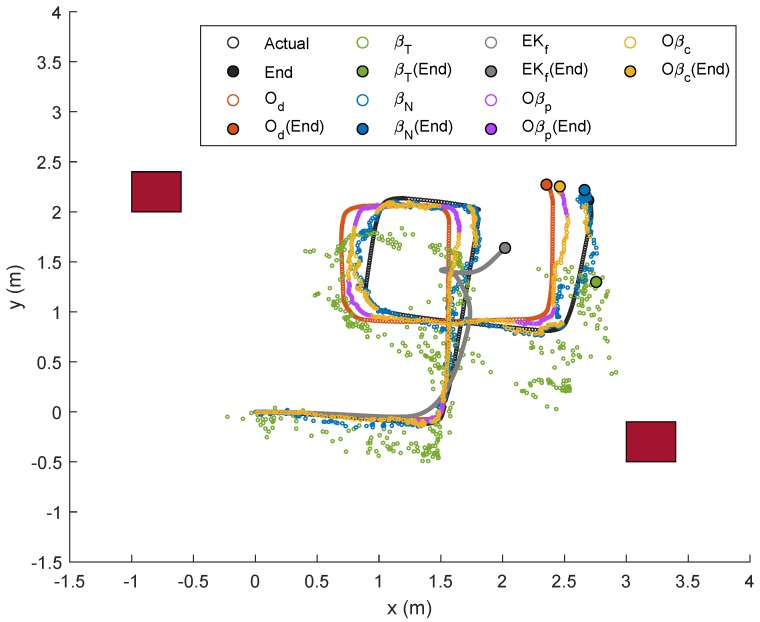
Localization of Node M in xy-plane in Trajectory 3.

**Figure 17:**
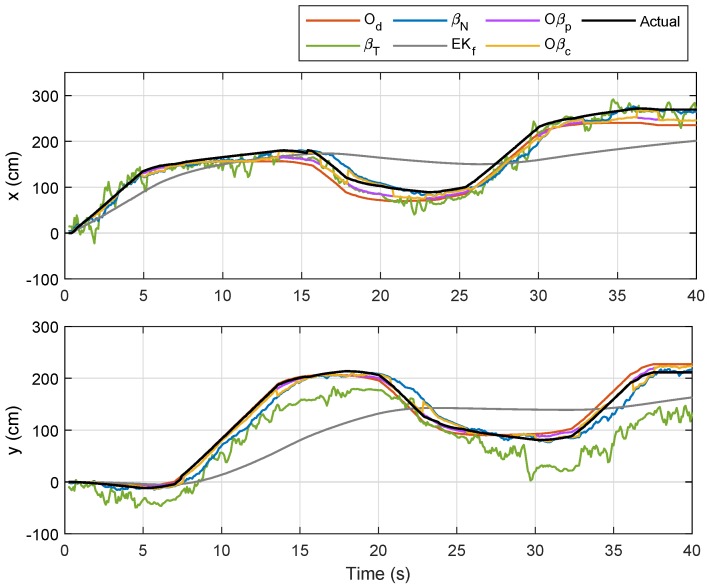
Localization of Node M in *x*- and *y*-directions in Trajectory 3.

**Figure 18:**
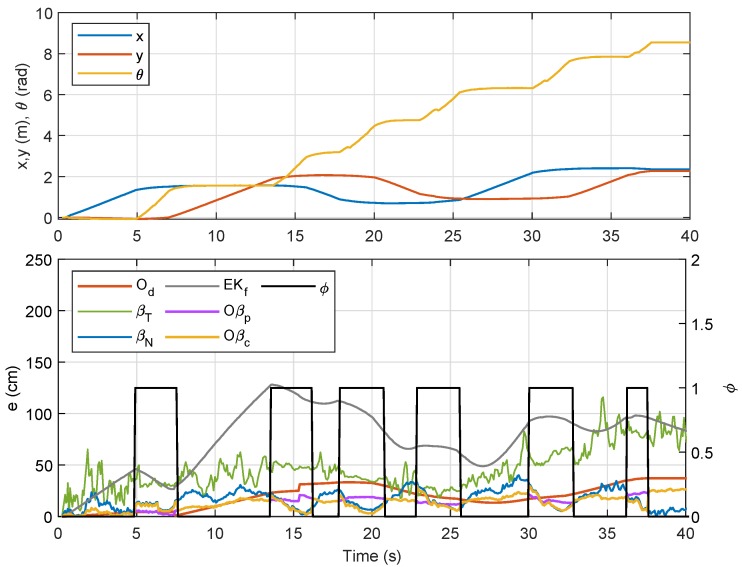
Trajectory 3: Position of NSBot2 in terms of x,y and θ based on odometry (**top**); and temporal localization-error analysis (**bottom**).

Another random trajectory with six linear motions alternating with six rotational motions was designed for Trajectory 3, and the corresponding localizations and error plots are illustrated in Figure 16, Figure 17 and Figure 18. In this case, the number of curves involved and the duration for rotational movement were relatively higher than those from the previous two trajectories and, as predicted, the approach via $O_{d}$ worsened as the error piled up after each turn. This is also shown numerically in Table 5, which presents a slightly different trend, where the error due to $O_{d}$ for each test was constantly higher than that from $\beta_{N}$. The proposed optimal sensor-fusion methods nevertheless led to the best performance, with error due to $O\beta_{c}$ being the least.

**Figure 19:**
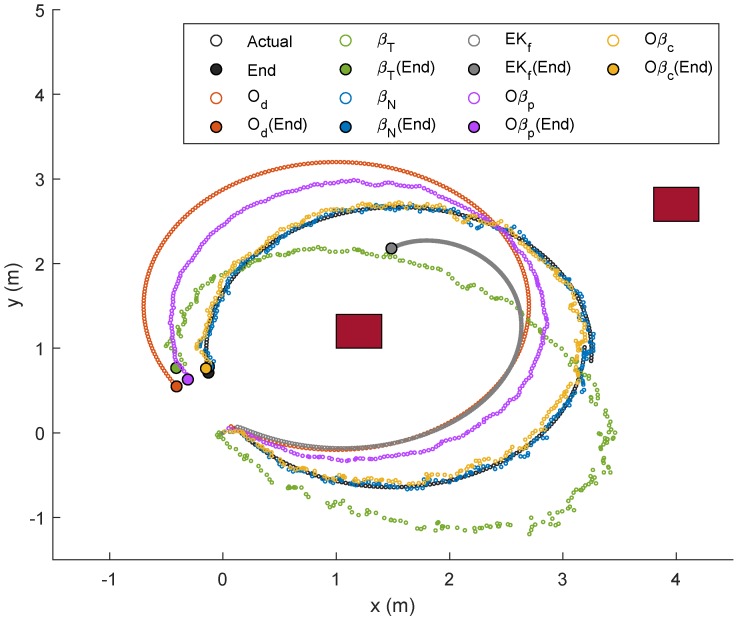
Localization of Node M in xy-plane in Trajectory 4.

**Figure 20:**
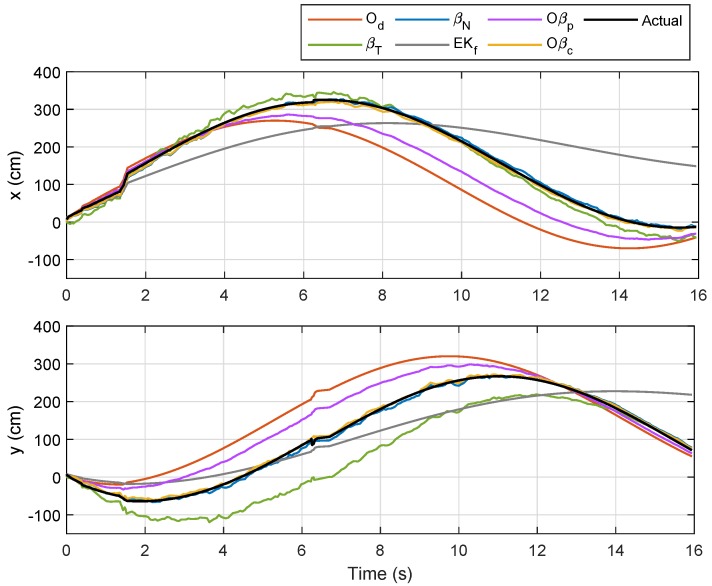
Localization of Node M in *x*- and *y*-directions in Trajectory 4.

**Figure 21:**
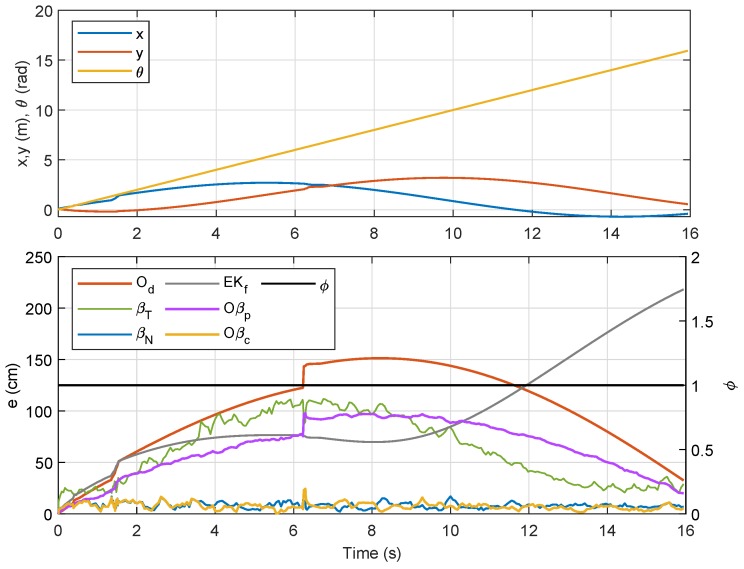
Trajectory 4: Position of NSBot2 in terms of x,y and θ based on odometry (**top**); and temporal localization-error analysis (**bottom**).

For the last trajectory, Trajectory 4, a continuous rotational motion was assigned to NSBot2 to form a circle-like path. The corresponding localizations and error plots are depicted in Figure 19, Figure 20 and Figure 21, while the numerical results are presented in Table 6. As can be seen in Figure 19, localizations based on $\beta_{N}$ and $O\beta_{c}$ were closer to the actual trajectory, whereas those from other methods clearly drifted. In this case, the trajectory via $O_{d}$ became much worse in comparison with the last three paths due to wheel slippage, and as ϕ=1 for $t\in [0,t_{f}]$, $O\beta_{p}$ prioritized $O_{d}$, which consequently led to a higher localization error. The numerical results for this last experiment are recorded in Table 6, which shows a significantly small error from $\beta_{N}$ as compared to those from $O_{d}$, $O\beta_{p}$, $EK_{f}$, and $\beta_{T}$. Interestingly, although $O_{d}$ led to a huge localization error in this scenario, the self-adaptive filtering method introduced in Corollary 1 resulted in a further improvement for each test, as highlighted in the last column of Table 6.

A summary of the average localization errors from the four experiments is presented in Table 7, along with total duration for rotational motion $\Delta t_{r}$ (in seconds and percentage, i.e., with respect to total execution time $t_{f}$). From the summarized numerical results, the performance via $O\beta_{c}$ was consistently the highest regardless of the value of $\Delta t_{r}$. On the contrary, the approach via $EK_{f}$ was the worst for all trajectories. This signifies that, while the extended Kalman filter usually results in a better performance particularly for sensor fusions, the error is likely to overshoot when localizing a mobile node at a certain speed range. This finding may be explained by the fact that a moving node leads to (possibly highly) nonlinear responses, as exemplified by the previous temporal analyses which could eventually make it unsuitable for localization via this approach. A closer inspection of $O_{d}$ and $\beta_{N}$ shows that the error via $O_{d}$ was lower than that via $\beta_{N}$ when $\Delta t_{r}$ was sufficiently small as can be observed in Trajectory 2. As $\Delta t_{r}$ became slightly higher, $\beta_{N}$ and $O_{d}$ became more competitive. Nevertheless, $\beta_{N}$ is advantageous when the mobile node is in full rotational movement, as revealed in the last experiment. From the table, it is also apparent that the proposed sensor-fusion techniques via $O\beta_{p}$ and $O\beta_{c}$ led to significant improvements for the first three trajectories, which involved rotational motions with durations of less than 37% of the total execution time. When the duration is sufficiently large, as typified by Trajectory 4, the performance can be further enhanced via $O\beta_{c}$. This desirable outcome is conclusively due to the optimization of μ in Corollary 1, which searches for the best error minimizer during the rotational motion.

## 4. Discussion and Conclusions

This study presents a new technique to improve indoor localization of a mobile node by utilizing Zigbee-based RSSI and odometry. In this work, a nonholonomic wheeled mobile robot was designed with capabilities to estimate its own location and movement from odometry data as well as RSSI values of four anchor nodes at once. As both localization methods suffer from their own limitations, this work contributes to a new methodological framework in which coordinates of the mobile node can more accurately be predicted by improving the path-loss propagation model and optimizing the weighting parameters for each localization technique prior to fusion. Simple convex searches are also introduced to find optimal error minimizers during the robot’s translational and rotational motions. In order to validate the proposed methods, several real-time experiments consisting of four different trajectories with different numbers of straight paths and curves, were carried out. Numerical and experiment results demonstrated that when both odometry data and RSSI values are available, Proposition 1 and Corollary 1 provide significant improvements to localization performance over the existing approaches. Corollary 1 in particular emerges as the best approach as it generalizes Proposition 1 and Lemma 1 with the additional self-adaptive filtering method, which autonomously optimizes the error minimizer when the rotational velocity within a specified range is detected.

While the proposed localization methods performed well in the considered scenarios, problems might arise when localizing a mobile node in buildings with multiple walls and moving objects. In this case, it is also expected that RSSI signals may be further degraded due to increased multipath effects. For future work, these conditions should be considered, and in order to alleviate effects from multipath interference, the proposed methods in the current work could be combined with other approaches, such as time of arrival (TOA), angle of arrival (AOA), as well as phase-shift measurement from anchor nodes. For localizing multiple mobile nodes, the proposed self-adaptive filtering approach could also be integrated with the compressive-sensing technique that only requires a small number of anchor nodes as demonstrated via simulations in [9]. The aforementioned methods may, however, exhibit different error patterns and behavior while the target node is moving; hence, modifications on the optimization techniques and the positions of the anchor nodes are required to maximize performance.

## Figures and Tables

**Table 1:** Numerical analysis on localization error from preliminary experiment.

Test	$E_{T}$ (Total)			$E_{T}$ When ϕ=1			$E_{T}$ When ϕ=0		
	$O_{d}$	$\beta_{T}$	$\beta_{N}$	$O_{d}$	$\beta_{T}$	$\beta_{N}$	$O_{d}$	$\beta_{T}$	$\beta_{N}$
1	820.2	2032.1	1195.3	232.8	439.6	87.2	580.8	1532.1	1014.3
2	825.0	2004.1	1045.1	233.9	479.6	97.0	591.1	1524.4	948.1
3	821.5	2221.4	1205.3	184.5	422.1	67.4	637.0	1799.3	1137.9
Average	**822.2**	2075.7	1101.1	219.3	457.0	**81.7**	**603.0**	1618.6	1033.4

**Table 2:** Analysis of localization error and optimal values of μ for Proposition 1 and Corollary 1.

Test	Optimal μ		$E_{T}$ (cms)	
	$\mu_{0}^{*}$	$\mu_{1}^{*}$	**Proposition 1**	**Corollary 1**
1	0.73	0.15	745.88	654.62
2	0.66	0.10	719.05	655.59
3	0.67	0.14	704.86	619.85
Average	0.69	0.13	723.26	643.35

**Table 3:** Localization error for Trajectory 1. The least error for each row is written in bold.

Test	$E_{T}$ (cms)					
	$\beta_{T}$	$\beta_{N}$	$O_{d}$	${\mathrm{EK}}_{f}$	$O\beta_{p}$	$O\beta_{c}$
1	1626.46	714.81	829.46	4812.55	567.82	**515.03**
2	1688.04	805.23	820.20	4797.16	559.83	**505.08**
3	1791.86	800.84	815.10	4596.16	561.28	**528.16**
4	1709.85	742.91	825.03	4809.81	551.35	**505.43**
5	1523.49	709.03	820.03	4775.22	545.40	**496.28**
Average	1667.94	754.56	821.96	4758.18	557.14	**510.00**

**Table 4:** Localization error for Trajectory 2. The least error for each row is written in bold.

Test	$E_{T}$ (cms)					
	$\beta_{T}$	$\beta_{N}$	$O_{d}$	${\mathrm{EK}}_{f}$	$O\beta_{p}$	$O\beta_{c}$
1	1669.58	771.25	693.48	4014.31	475.44	**432.16**
2	1797.22	658.41	787.65	3918.32	524.67	**472.28**
3	1754.16	836.44	720.32	3812.33	522.02	**474.16**
4	1656.05	672.98	710.22	3772.11	500.73	**442.78**
5	1841.64	845.08	699.23	3645.27	542.61	**479.06**
Average	1743.73	756.83	722.18	3832.47	513.09	**460.09**

**Table 5:** Localization error for Trajectory 3. The least error for each row is written in bold.

Test	$E_{T}$ (cms)					
	$\beta_{T}$	$\beta_{N}$	$O_{d}$	${\mathrm{EK}}_{f}$	$O\beta_{p}$	$O\beta_{c}$
1	1856.12	654.20	782.06	3008.95	573.20	**522.96**
2	1841.73	726.68	780.11	3010.13	596.72	**543.36**
3	1916.94	746.36	795.12	3040.33	582.61	**531.27**
4	1836.61	675.28	786.34	3029.90	559.14	**503.21**
5	1879.49	644.47	795.12	3040.33	583.79	**510.86**
Average	1866.18	689.40	787.75	3025.93	579.09	**522.33**

**Table 6:** Localization error for Trajectory 3. The least error for each row is written in bold.

Test	$E_{T}$ (cms)					
	$\beta_{T}$	$\beta_{N}$	$O_{d}$	${\mathrm{EK}}_{f}$	$O\beta_{p}$	$O\beta_{c}$
1	995.60	133.49	1600.46	1547.17	1014.26	**117.60**
2	980.29	127.60	1598.23	1501.31	1017.25	**124.19**
3	990.35	149.62	1498.61	1487.52	1018.15	**132.50**
4	1003.9	136.12	1599.17	1523.21	1011.81	**111.10**
5	1004.0	131.40	1587.87	1500.23	1018.33	**128.50**
Average	994.83	135.65	1576.87	1511.89	1015.96	**122.78**

**Table 7:** Summary of average localization errors for Trajectories 1–4.

Trajectory	Rotational Motion		Average $E_{T}$ (cms)					
	$\Delta t_{r}$ (s)	$\Delta t_{r}/t_{f}$ (%)	$\beta_{T}$	$\beta_{N}$	$O_{d}$	${\mathrm{EK}}_{f}$	$O\beta_{p}$	$O\beta_{c}$
1	10.39	26.0	1667.94	754.56	821.96	4758.18	557.14	**510.00**
2	8.87	22.2	1743.73	756.83	722.18	3832.47	513.09	**460.09**
3	12.92	36.9	1866.18	689.40	787.75	3025.93	579.09	**522.33**
4	16.00	100	994.83	135.65	1576.87	1511.89	1015.96	**122.78**

## Notations and Abbreviations

The following notations and abbreviations are frequently used in this manuscript:

WSN	wireless sensor network
GPS	global positioning system
RSSI	received-signal-strength indicator
γ	path-loss exponent coefficient
v,ω	translational velocity, rotational velocity
$\omega_{r},\omega_{l}$	right-wheel speed, left-wheel speed
x,y,θ	NSBot2’s position in *x*- direction, *y*- direction, and heading angle
$t_{s}$	sampling time (in odometry)
μ	weighting parameter
$\mu_{0}^{*},\mu_{1}^{*}$	optimal weighting parameters (error minimizer when ϕ=0, error minimizer when ϕ=1)
*e*	transient error (in cm)
$E_{T}$	total error (in cms)
ϕ	indicator for rotational motion
$O\beta_{N}$	$E_{T}$ based on Proposition 1/Corollary 1 (only in Section 2.4)
$\beta_{T}$	localization via Zigbee-based RSSI method with theoretical path-loss model and a moving average filter in Equation (19)
$\beta_{N}$	localization via Zigbee-based RSSI method as described in Lemma 1
$O_{d}$	localization via odometry
$EK_{f}$	localization via extended Kalman filter
$O\beta_{p}$	localization via Proposition 1
$O\beta_{c}$	localization via Corollary 1
$t,t_{f},\Delta t_{r}$	time, execution time, total duration of rotation (in seconds)
